# Theoretical Analysis of Riboflavin Adsorption on Hexagonal Boron Nitride for Drug Delivery Applications: Unveiling the Influence of Point Defects

**DOI:** 10.3390/ijms241411648

**Published:** 2023-07-19

**Authors:** Liubov Yu. Antipina, Kristina Yu. Kotyakova, Pavel B. Sorokin

**Affiliations:** 1 Laboratory of Inorganic Nanomaterials, Research Center of Inorganic Nanomaterials, National University of Science and Technology “MISIS”, Leninsky Prospect 4, 119049 Moscow, Russia; pbsorokin@misis.ru; 2 Research Center of Inorganic Nanomaterials, National University of Science and Technology “MISIS”, Leninsky Prospect 4, 119049 Moscow, Russia; kristinkagudz@mail.ru

**Keywords:** boron nitride, riboflavin, DFT calculations, adsorption, vacancy defect, drug delivery

## Abstract

This research delves into the intriguing realm of investigating the stability of vitamin B2 (riboflavin, Rf) on hexagonal boron nitride (h-BN), both in its pristine state and in the presence of vacancy defects, with the aim of harnessing their potential as carriers for drug delivery applications. Employing the density functional theory (DFT), we perform binding energy calculations and analyze the electronic structure of the BN@Rf system to unravel the nature of their interactions. Our comprehensive DFT calculations unequivocally demonstrate the spontaneous physical sorption of the drug onto the h-BN surface, facilitated by the formation of π-π stacking interactions. The adsorption energy spans a range from −1.15 to −4.00 eV per system, emphasizing the robust nature of the BN@Rf bonding. The results show that the HOMO and LUMO of riboflavin are located exactly in the region of the iso-alloxazine rings of riboflavin. This arrangement fosters the formation of π-π stacking between riboflavin and boron nitride, effectively facilitating the transfer of electron density within the BN@Rf system. Furthermore, our investigations reveal the significant impact of vacancy defects within the boron nitride lattice. These vacancies alter the behavior of the structure, prompting riboflavin to metamorphose from an electron donor to an electron acceptor, expanding our understanding of the interplay between boron nitride defects and riboflavin sorption. Therefore, it is imperative to exert meticulous oversight of the structural integrity of h-BN, given that the existence of vacancies may lead to a noticeable change in its adsorption properties. The obtained data could amplify our capacity to conceive and refine drug delivery h-BN-based systems.

## 1. Introduction

Modern bioengineering research focuses on developing new therapies and drug delivery approaches. The acidic environment of the gastrointestinal tract can render drugs ineffective before they reach their target cells, making oral delivery a challenging method [1]. To enhance therapy effectiveness and reduce the body’s burden, direct delivery of anticancer drugs to the affected sites is essential. However, most drugs lack the ability to target specific cancer cells, requiring the development of safe carriers and targeted approaches. Targeted cancer therapy can employ specific receptors and transporters, such as riboflavin [2,3], glucose [4], transferrin [5], etc., which are highly overexpressed on some cancer cells’ surfaces. These target sites can be utilized to achieve more effective and efficient cancer treatment.

The bioavailability of drugs for tumor tissues is relatively low and higher doses are required, leading to increased toxicity in normal cells and an increase in the development of multidrug resistance. Compared to systemically delivered drugs, nanoscale drug carriers have shown potential by increasing treatment efficacy while avoiding toxicity in normal cells as a result of features such as their highly selective accumulation in tumors due to their enhanced permeability and retention effects and active cellular uptake [6].

Riboflavin (Rf), also known as vitamin B2, is a water-soluble vitamin that plays a vital role in human health. It serves as the primary biochemical source of the flavin group in cells, readily forming flavinmononucleotide and flavinadenindinucleotide. Rf is involved in various cellular processes, including the metabolism of lipids, ketone bodies, carbohydrates, and proteins [7,8]. In pathological states, including oncogenesis, the metabolism and ingestion of Rf are increased, sometimes at the expense of riboflavin entry into other tissues [9]. It was found that the concentration of Rf carrier protein in the cancerous lesion is markedly increased for people with breast cancer [10]. Moreover, riboflavin accumulation in human breast cancer cells is specific and receptor-mediated [11], making it more efficient than folic acid [12]. An insufficient amount of Rf in the body can lead to the development of seborrheic dermatitis, changes in the skin of the scrotum and vulva, cheilosis, and anemia [13,14]. However, the human body cannot synthesize Rf, and it must be obtained from food sources, particularly liver, cereals, bakery products, muscle meats, and eggs [15].

This requires the targeted delivery of riboflavin to the body, which can be achieved using nanoparticle carriers. These nanoparticles must be biocompatible, have a high specific area, and at the same time able to desorb the drug when delivered to the desired area. These requirements are satisfied by nanomaterials having a layered sp^2^-hybridized structure [16,17]. Moreover, the presence of a π-conjugated isoalloxazine residue in the riboflavin (Rf) molecule facilitates its binding to 2D materials that possess a π-conjugated structure, such as graphene [8,18] or single-walled carbon nanotubes (SWCNTs) [19,20]. The flat isoalloxazine structure promotes intermolecular interactions with nanomaterials through π-π interactions, leading to the organized assembly of riboflavin molecules on the surface of carbon nanomaterials in an ordered manner [19,20,21]. Furthermore, flavin mononucleotide phosphate can act as a stabilizer of aqueous dispersions of graphene [22]. These π-π stacking interactions also play a role in the interaction of flavin mononucleotide with the hydrophobic surfaces of boron nitride nanotubes (BNNTs) [23,24].

This study aims to investigate the theoretical interactions between riboflavin and hexagonal boron nitride (BN), which has been identified as a promising platform for drug delivery [25,26,27]. Previous research has demonstrated that BN is an effective adsorbent for various drugs, with hexagonal BN mesoporous fibers [28], h-BN porous whiskers [29], cotton flower-like porous BN [30]^,^ and stamen-shaped porous boron carbon nitride nanoscrolls [31] exhibiting high sorption capabilities for different organic compounds. In addition, BN nanoparticles have been proposed as a bio-compatible drug delivery system with pH-sensitive release of therapeutic agents for targeting cancerous tumors [32,33,34], delivery of anticancer drugs to tumor cells [35], and even their destruction [36]. Recent studies have indicated that BN nanoparticles at concentrations below 100 mg/L can be safely used as a nanomaterial in medical applications [37,38].

In this study, we theoretically considered hexagonal boron nitride as a potential carrier for the vitamin B2 molecule. It has been shown that perfect BN strongly affects the energy and nature of Rf binding. The analysis of the charge density and the interpretation at the electronic level of the adsorption of Rf on BN has shown that, depending on the type of vacancy defect, Rf can be both an electron density donor and an acceptor. The electron density is transferred to the drug-carrier system via π-π stacking. This changes the effective charge Rf and the carrier.

## 2. Results and Discussion

### 2.1. Molecular Geometry and Binding Energies

**Figure 1:**
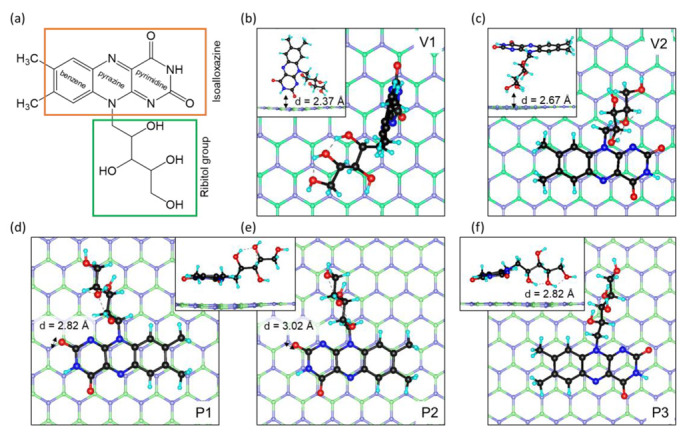
(**a**) Structure of riboflavin with highlighted main functional groups. (**b**–**f**) Optimized structure of riboflavin on BN in different positions. The boron, nitrogen, carbon, oxygen, and hydrogen atoms are marked in green, blue, black, red, and cyan. The insets show the side views of these structures; for (**d**,**e**) the side views are similar.

In order to determine the most stable configuration of a riboflavin molecule (Rf) on perfect hexagonal boron nitride (BN) (BN@Rf) or boron nitride with a boron or nitrogen vacancy (BN(Bv) or BN(Nv), respectively), the Rf was placed above the carrier in five different orientations: two vertical positions (V1 and V2), and three planar positions (P1, P2, and P3), as depicted in Figure 1. A comparison of the geometries before and after optimization reveals that there is no reorientation observable in the drug-carrier complexes. A comparison of the geometries before and after optimization reveals that there is no reorientation observable in the drug-carrier complexes. The change in bond lengths and angles in the vitamin B2 molecule does not exceed 1%. The position relative to the boron nitride also does not change significantly. The greatest changes with respect to the interposition of the drug and the carrier are observed in the case of the vacancies. Ribitol residue oxygen is attracted to the broken bonds on the vacancy, closing it, and the distance between the riboflavin and the BN surface decreases.

The adsorption energies of the drug-carrier complexes for each position were calculated as follows:

(1) $$E_{\mathrm{ads}}=E_{\mathrm{tot}}-E_{\mathrm{BN}}-E_{\mathrm{rib}},$$

where E_tot_ is the total energy of the system and E_BN_ and E_rib_ are the energies of a freestanding carrier and a riboflavin molecule, respectively. All the binding energies for the different configurations (Table 1) are negative, and adsorption clusters are more stable than freestanding structures; therefore, adsorption on h-BN is an exothermic and spontaneous process. According to the calculated adsorption energies, it can be concluded that there is a relatively strong non-chemical interaction between the drug molecule and all types of carriers.

According to the adsorption energies data, the most favorable positions of riboflavin on all types of the boron nitride surface is the planar position (P1, P2, and P3) with π-π vertical stacking, when a six-membered ring of riboflavin isoalloxazine residue prefers a parallel orientation with respect to the h-BN surface. This observation indicated that a π-π stacking interaction with vertical distances ~2.4–3.0 Å between the drug and the carrier is formed. In addition, for the P1 and P2 configurations, when the ribitol residue is oriented away from the boron nitride surface, the stacking occurs vertically, hexagonal over hexagonal. In the case of the P3 configuration, when the ribitol residue is directed onto the h-BN surface, there is a shift in the isoalloxazine residue site, so that half of the atoms are on top while the other half are at the hollow sites directly above the centers of the hexagonal rings of the h-BN layer. This probably leads to the disruption of the π-π stacking interaction and, therefore, less bonding energy. The most stable among the three vertical stacking configurations is P2, when the three nitrogen atoms of isoalloxazine residue are above the nitrogen atoms of h-BN (P2), rather than the boron atoms (P1). In the case of the defects in the plane positions, there is also a slight shift in the isoalloxazine residue site relative to the surface of the boron nitride, so the functional atoms of the pyrimidine ring are located above the defective region, increasing the stability of the BN(Bv)@Rf and BN(Nv)@Rf systems.

It should be noted that in the case of riboflavin adsorption in vertical positions V1 and V2 on the boron nitride in the presence of a nitrogen vacancy (BN(Nv)@Rf), when one of the oxygen atoms of the molecule of riboflavin is directed strictly to the defect area, the formation of a chemical bond B-O is observed. However, this generates sufficiently large distortions in the structure of planar boron nitride, and, in the case of position V2, there is also a detachment of the proton from the OH group. Such distortions increase the energy of the system and, therefore, the adsorption energy, which makes these structures less favorable compared to the plane configuration (P1, P2, P3).

A comparison of the geometries of the most stable P2 configurations before and after optimization shows that the drug-carrier complexes BN@Rf show no appreciable change in geometry. The change in the bond lengths of riboflavin relative to the free-standing molecule in the case of its adsorption on BN and BN(Bv) does not exceed 0.02 Å. The C-C bonds in the molecule become a little shorter, but the C-OH bonds are elongated (up to 0.02 Å). In the case of BN(Nv), the strongest changes in bond length (up to 0.05 Å) are observed in the region of the pyrimidine ring of the isoalloxazine site; the C-C bonds also become shorter, but the C-N bonds are lengthened, which leads to some deformation of the structure. Similar changes in the structure of riboflavin occur during interaction with carbon nanostructures [20], which confirms the fact of a similar interaction through π-π stacking. Comparing the obtained adsorption energies from the previous research [39,40,41,42] and the calculated E_ads_ in our research reveals that the h-BN have good potential for the delivery of riboflavin and other drugs. 

### 2.2. Charge Analysis

**Figure 2:**
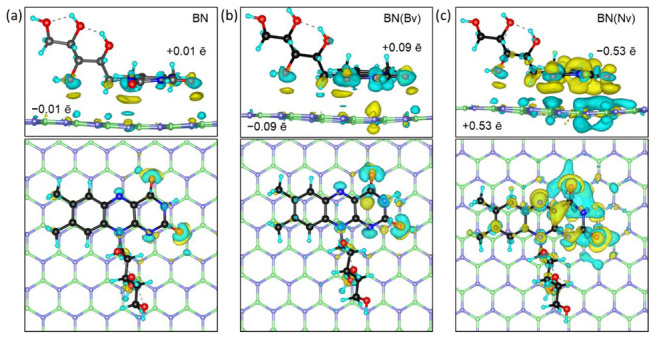
Distribution of the spatial charge density difference in (**a**) BN@Rf, (**b**) BN(Bv)@Rf, and (**c**) BN(Nv)@Rf structures and the corresponding freestanding parts, side and top view. The loss and gain of charge are denoted by blue and yellow clouds, respectively. The boron, nitrogen, carbon, oxygen, and hydrogen atoms are marked by green, blue, black, red, and cyan colors, respectively. The isosurface constant value is 0.001 eV/Å.

For the further analysis of riboflavin binding to boron nitride, we chose the most stable P2 configuration, which has the highest binding energy (Table 1). In order to understand the nature of the riboflavin interaction with a BN carrier, the difference in the spatially distributed electron density between the whole system and each separate part, hexagonal boron nitride and the vitamin molecule, is presented in Figure 2. 

During the interaction of riboflavin with the defect-less surface of boron nitride (BN@Rf) there is no significant interaction. The redistribution of the electron density is rather weak and the total electron transfer from riboflavin to boron nitride, calculated by Bader analysis, is ~0.01 ē; boron nitride acquires a weak negative charge, and the molecule of riboflavin is weak positive. The main electron transfer is observed in the oxygen region of the pyrimidine ring, with a transfer of 0.02 ē from oxygen to the underlying nitrogen of the boron nitride, and in the ribitol group from the surface of the boron nitride to the oxygen-OH group of the alcohol. In addition, due to the conjugated bonding system of isoalloxazine rings, electron density to oxygen atoms is pulled not only from the boron nitride but also from the conjugated system of riboflavin itself, with carbon atoms and hydrogen atoms acquiring a positive charge (Figure 2a).

In the case of the boron vacancy (BN(Bv)@Rf), the area of charge redistribution remains the same, but the total charge of the systems increases up to −0.09 ē on BN(Bv) and +0.09 ē on riboflavin (Figure 2b).

The nitrogen vacancy leads to a rather strong redistribution of the electron density in the vacancy region and in the riboflavin as a whole (Figure 2c). A rather strong positive charge (total +0.21 ē per three boron atoms) is generated in the vacancy region due to the presence of broken bonds on the boron atoms, also involving nearby atoms of h-BN (+0.14 ē per five nitrogen atoms surrounding the boron atoms). The oxygen atoms of the pyrimidine ring of the isoalloxazine residue pull on themselves a total of −0.14 ē, and a sufficient number of electrons also flow through conjugated bonds to the rings of the isoalloxazine residue. The redistribution of the electron density in the region of the ribitol residue remains the same as in the case of a BN@Rf. In summary, this leads to the fact that, in the presence of a nitrogen vacancy, boron nitride is positively charged (+0.53 ē) and riboflavin is negatively charged (−0.53 ē). 

This can be explained in terms of the electronegativity of the nitrogen and boron atoms. In the case of the nitrogen vacancy BN(Nv), boron, as a less electronegative element (X_Pauling_(B) = 2.04), more easily gives unpaired electrons to the conjugated system of riboflavin, while the uncompensated nitrogen bonds (X_Pauling_(N) = 3.04) for BN(Bv) pull the electron density on itself. 

### 2.3. Orbital Analysis

**Figure 3:**
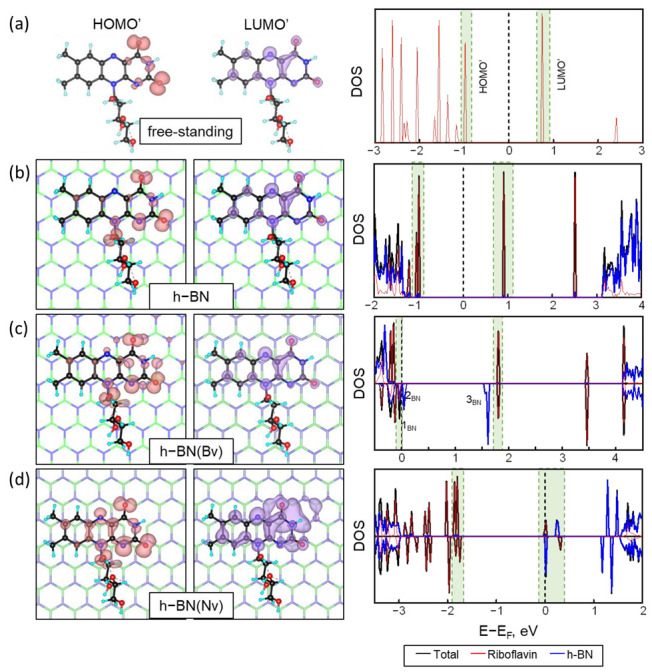
The isosurface of the HOMO’ and LUMO’ for (**a**) free-standing riboflavin, (**b**) BN@Rf, (**c**) BN(Bv)@Rf, and (**d**) BN(Nv)@Rf structures and corresponding density of states (black line), resolved per riboflavin molecule (red line) and corresponding carrier (blue line). The isosurface constant value is 0.005 eV/Å. The green dashed lines on the DOS panels highlight the regions of the corresponding orbital. The Fermi level is taken as zero and shown as a black dashed line.

The density of states (DOS) plots are important for understanding the electronic properties and changes during interactions. The partial density of states plots were obtained for BN, BN(Bv), and BN(Nv) nanosheets with an adsorbed riboflavin molecule in the most stable position, P2 (Figure 1e). From the DOS of a freestanding molecule, the positions of the HOMO and LUMO orbitals can be determined. The Kohn–Sham density functional theory (DFT) [43,44], implemented in Siesta [45], serves as the established method for determining electronic states and orbital energy levels. Initially, Kohn–Sham orbitals were regarded as lacking physical significance, but extensive research has demonstrated their practical utility in orbital analysis [46,47]. Moreover, it has been established that the first ionization potential corresponds to the absolute value of the HOMO [48,49]. Unfortunately, although DFT has a physically sound basis, at least for HOMO, approximate exchange-correlation functionals do not always guarantee an accurate calculation of orbital energy levels [50]. Local density approximation (LDA) and generalized gradient approximation (GGA) exchange-correlation functionals, such as PBE, strongly underestimate HOMO-LUMO energy gaps. Moreover, for a structure in periodic boundary conditions, such as h-BN, it is not applicable to talk about molecular orbitals directly. However, it is possible to analyze the energy levels near the Fermi energy and relate these to the HOMO/LUMO of a free-standing molecule [51,52]. Thus, instead of directly analyzing molecular orbitals, in the following analysis, we denote the levels near the Fermi energy for BN@Rf systems as HOMO’ and LUMO’, because these are not molecular orbitals by themselves, but electronic levels. On Figure 3 the orbitals are represented by a common isosurface without being divided into positive or negative phases of the orbitals.

For the freestanding riboflavin molecule, HOMO is located on the pyridine ring of the isoalloxazine residue, while LUMO is more evenly distributed over the whole isoalloxazine residue (Figure 3a). This arrangement of the orbitals is the reason why the π-π vertical stacking configuration, where the overlapping area of the electron clouds will be maximal, is the most stable configuration. 

From the PDOS and the redistribution of the corresponding energy levels, we can see that, in the case of perfect h-BN, occupied and unoccupied molecular states of riboflavin fall in the BN energy gap. Therefore, there is no overlap between the orbitals of riboflavin and boron nitride. The electronic levels near the Fermi energy (HOMO’ and LUMO’) are almost totally related to the riboflavin molecule, and there is no mixing of energy levels. At the same time, the HOMO’ is slightly changed relative to the HOMO of the freestanding molecule. The HOMO’ is redistributed to the whole region of the isoalloxazine residue and slightly to the first oxygen of the ribitol residue. LUMO’ remains unchanged for all cases (Figure 3b). 

The introduction of vacancy defects in the structure of boron nitride leads to the formation of spin splitting due to the presence of unpaired electrons of broken bonds [53,54]. 

During the adsorption of riboflavin onto a BN(Bv) carrier, the HOMO’ and LUMO’ of riboflavin do not change their shape significantly (Figure 3c). However, there is a slight overlap of the HOMO’ region with the boron nitride levels in the defect area. Near the Fermi level, impurity levels of boron nitride (1_BN_, 2_BN_, 3_BN_, Figure 3c) appear due to uncompensated bonds on the nitrogen. Moreover, these levels are attributable only to spin-down states. Among these three boron nitride levels, only 1_BN_, which lies below the Fermi level, forms an overlap with riboflavin’s electron-donating HOMO’. The HOMO’ shows little redistribution with weak overlap to the region of the isoalloxazine residue and the ribitol edge. This overlap of riboflavin’s HOMO’ with the levels of boron nitride turns the adsorbed molecule into an electron donor, as was shown in the Bader charge calculation (Figure 2b). Two other levels (2_BN_ and 3_BN_), lying above the Fermi level, do not interact with the adsorbed molecule. The LUMO’ level of the riboflavin molecule does not change.

In the case of a nitrogen defect in the DOS of the BN(Nv)@Rf system, an energy level below the Fermi level, formed purely by the contribution of the riboflavin molecule, shows no overlap with the energy levels of boron nitride (Figure 3d). This level can be attributed to the HOMO’ of the riboflavin molecule, but it overlaps with the lower energy level of riboflavin. This leads to this level covering the pyrimidine and pyrazine rings wholly. The clouds on the ribitol residue change slightly, as in the case of BN(Bv)@Rf. The two levels with upper energy for spin-up and spin-down represent the LUMO’ split spin orbital. Moreover, one of them is located directly at the Fermi level, and the other one is slightly higher. This splitting LUMO’ overlaps with the impurity levels of boron nitride arising from the presence of a vacancy. This overlapping leads to a weak redistribution of the LUMO’ orbital in the pyrimidine ring region. From the obtained overlap of orbitals it can be concluded that during the adsorption of riboflavin on the BN(Nv) carrier, in contrast with BN and BN(Bv), it will be an electron acceptor, whereas the boron nitride will be a donor of electrons, which explains the transfer of electron density on 0.5 ē to the riboflavin molecule (Figure 2). 

## 3. Materials and Methods

The theoretical analysis of the atomic structure and stability of the riboflavin-BN nanosystems were performed using density functional theory (DFT) [43,44] within the generalized gradient approximation (GGA) using normalized Troullier-Martins [55] pseudopotentials in the SIESTA software package [45]. The plane-wave energy cutoff was set to 200 Ry. To calculate the equilibrium atomic structures, a Brillouin zone of 4 × 4 × 1 was chosen according to the Monkhorst-Pack scheme [56]. To calculate the electronic properties, the cutoff of the k-grid was 16 × 16 × 1. The van der Waals interactions caused by geometry optimization were taken care of by the VDW-DRSLL correlation energy functional [57,58].

We designed hexagonal boron nitride structures containing 112 atoms in the orthorhombic supercell (B_56_N_56_). The calculated lattice parameter for a unit cell of h-BN was 2.515 Å, which corresponds very well with the experimental data (2.51 Å [59]). After point defects and riboflavin adsorption were introduced, the lattice was also optimized to account for cell changes. The lattice changes after introducing the defects were less than 1%. The introduction of riboflavin into the system had practically no effect on the lattice parameter. The systems were modeled as supercells with a sufficiently large vacuum gap (at least 15 Å) to neglect intermolecular interactions in the non-periodic direction.

## 4. Conclusions

This study employed density functional theory (DFT) simulations to comprehensively investigate the adsorption behavior of riboflavin (Rf) on both defect-free and vacancy-containing hexagonal boron nitride (h-BN) systems. The findings reveal that the Rf molecule undergoes physical adsorption on the surface of the carrier, exhibiting minimal alteration in its chemical structure.

The most stable configuration observed involves the parallel alignment of the riboflavin molecule with the h-BN surface, forming π-π stacking interactions. This is corroborated by the adsorption energies obtained at various positions of the drug molecule. The molecular orbitals of riboflavin’s isosurfaces provide a comprehensive understanding of the binding nature between riboflavin and h-BN based on the HOMO and LUMO location on the isoalloxazine site.

Remarkably, the presence of nitrogen vacancies significantly impacts the binding characteristics, as the carriers interact with the vacant riboflavin orbitals. Consequently, riboflavin transforms into an electron acceptor in the BN(Nv)@Rf system, attracting electron density by approximately 0.5 ē. This behavior starkly contrasts with the interactions observed in defect-free h-BN and h-BN with boron vacancies (BN(Bv)).

These results validate the potential of h-BN as a promising carrier for riboflavin molecules, as the π-π bond formed between the drug and the carrier exhibits substantial strength, providing a solid foundation for drug delivery systems. However, it is crucial to exercise control over the structural perfection of h-BN, as the presence of vacancies can induce charging on riboflavin. 

In summary, our study provides valuable insights into the stability and interactions of vitamin B2 with hexagonal boron nitride. The comprehensive understanding gained regarding their binding characteristics and the influence of defects enhances our ability to design and optimize drug delivery systems based on h-BN.

## Figures and Tables

**Table 1:** The adsorption energies (eV) of riboflavin with the BN surface in different configurations.

Configuration	Carrier Types		
	BN	BN(Bv)	BN(Nv)
V1	−1.82	−1.99	−3.64
V2	−1.15	−2.22	−2.77
P1	−3.49	−3.55	−3.69
P2	−3.73	−3.93	−4.01
P3	−3.16	−3.10	−3.28

## Data Availability

Not applicable.
